# The University of London

**Published:** 1907-03-23

**Authors:** 


### THE UNIVERSITY OF LONDON.

### THE SENATORIAL ELECTION IN THE FACULTY OF MEDICINE.

The result of the election of two representatives of the Faculty of Medicine upon the Senate of the University of London on Tuesday last will probably have an important effect upon the policy of the University with reference to the teaching of the Preliminary and Intermediate subjects of the medical curriculum. The election was contested, the policy of the University with reference to the so-called "Concentration" scheme being attacked by Dr. Caley (of St. Mary's Hospital) and Mr. Leonard Hill (of the London Hospital). Considerable interest was aroused by the contest, and circulars and fly-leaves were published broadcast by the candidates on both sides. At a very full meeting of the Faculty Professor Rose Bradford and Dr. Kingston Fowler—the previous representatives of the Faculty upon the Senate and advocates of the policy of "Concentration" in the form of three centres, University College, King's College, and a third centre at South Kensington—were defeated, and Drs. Caley and Hill elected in their place. This apparent reversal of the previous policy of the Faculty with reference to the concentration of the early medical studies was somewhat unexpected, though to those familiar with the conditions prevailing at the larger medical schools it had been ripening for some time past.

The policy of Concentration was first proposed to the schools by the University in 1901, and, in response to inquiries, the schools generally expressed opinions in favour of such concentration of the early studies at one centre or at as few centres as possible.

The vision in the minds of the members of the University who first advocated the scheme was the foundation of one great centre in some central district of London which should be large enough to embrace all the medical students in the metropolis, should be neutral to all the schools and favouring none, and that by endowment and organisation giving an education worthy of the greatest city in the world. This was a grand conception and attracted the sympathy of nearly all the schools, thought it was apparent even then to some of the more efficient and prosperous that they did not stand to gain by such a process of equalisation, but rather to lose. It was felt, however, that some sacrifice was worth making for such a comprehensive change, and the Faculty of Medicine, as well as the individual schools, advocated the active prosecution of this policy.

Times have changed since then, and Concentration has not developed upon these broad lines. The University has incorporated University College and made it a centre for the teaching of the early studies, and the same course has been adopted at King's College, which is also shortly to be incorporated. Already two centres have thus been created, and the University proposes, in addition, to build yet another centre—possibly at South Kensington—for which about £70,000 has been obtained, intending to make a start when a sum of £100,000 is available. The original idea, therefore, of one centre worthy of London and its educational opportunities has faded into thin air, and though the Faculty has never discussed the subject again, the individual schools, whose interests are likely to be affected, are face to face with a condition of things which they did not anticipate in 1901. In addition to the multiplication of the number of centres, another factor has entered into the policy of the University, and that is the institution of internal examinations at their three centres; or, in other words, the students will be given the advantage of being examined by their own teachers, whereas students from other schools would have to submit to an examination conducted upon the present or external lines. Whether internal examinations are educationally of great value is open to discussion; but it is quite certain that, rightly or wrongly, the idea would get abroad among the students that it would be easier to pass from a University centre than from a school not similarly favoured, and, human nature being what it is, the competition among the three University centres would probably tend to a gradual deterioration from the uniformly high standard which has always been the glory of the degrees of the University of London. Such being the policy of the University, it was obvious to the schools that if they permitted it to proceed with its third centre, take up its option of a site in South Kensington, build and start an Institute upon the lines indicated above, with internal examinations and all the advantages of University support, their extinction would be inevitable and only a matter of time. In 1901 the University stated that when the time was ripe the schools would be again consulted and terms of adhesion discussed. On the present occasion Professor Rose Bradford and Dr. Fowler stated in their election address that they did not consider that such time had yet arrived. There was a widespread idea that if the schools did not express their opinion now it would be too late for them to protest when their opinion was asked by the University, and the election of Drs. Caley and Hill is the answer of the schools, through the Faculty, to the policy of the University.

It is difficult to see how the result could have been otherwise, for the most ardent reformer could hardly expect the medical schools to assist in a scheme which was certain to affect them most seriously from a financial point of view without really improving medical education in London. With the moneys at the disposal of the University for the purpose of their Institute of Medical Sciences at South Kensington a centre could not be built which would be any more capacious or efficient than the three great medical schools east of Temple Bar, and of these both St. Bartholomew's and the London Hospitals have publicly expressed their determination not to participate in any such scheme as that now proposed. For other smaller schools who may wish to relieve themselves of the burden of the purely scientific portion of the curriculum there is still ample accommodation at University and King's Colleges, without the expenditure of any further sums upon bricks and mortar.

The result of the election of the Faculty of Medicine does not signify that there is any widespread spirit of reaction abroad, or that the University should cease its efforts towards the improvement of medical education. If the Senate is wise it will take a suggestion from the present course of events, abandon the idea of internal examinations as being possibly detrimental to the credit of its medical degrees, and expend its energies and funds in the assistance of the efficient medical schools by grants in aid, and in the development, of education and research in the more strictly medical branches of the curriculum, both in the schools and in its own laboratories.

